# Patterns of Distant Metastasis Between Histological Types in Esophageal Cancer

**DOI:** 10.3389/fonc.2018.00302

**Published:** 2018-08-08

**Authors:** San-Gang Wu, Wen-Wen Zhang, Jia-Yuan Sun, Feng-Yan Li, Qin Lin, Zhen-Yu He

**Affiliations:** ^1^Department of Radiation Oncology, Xiamen Cancer Hospital, First Affiliated Hospital of Xiamen University, Xiamen, China; ^2^State Key Laboratory of Oncology in South China, Department of Radiation Oncology, Sun Yat-sen University Cancer Center, Collaborative Innovation Center of Cancer Medicine, Guangzhou, China

**Keywords:** esophageal cancer, metastasis, histological type, population-based cancer registry, epidemiology

## Abstract

**Introduction:** Distant metastasis remains the major cause of treatment failure in esophageal cancer, though there have been few large-scale studies of the patterns of distant metastasis in different histological types. We investigated the patterns of distant metastasis in esophageal adenocarcinoma (AC) and squamous cell carcinoma (SCC) using a population-based approach.

**Methods:** Patients with *de novo* stage IV esophageal cancer at diagnosis were identified using the Surveillance, Epidemiology, and End Results database. Multivariable logistic regression was performed to identify potential risk factors for site-specific distant metastasis to the distant lymph nodes, bone, liver, brain, and lung at diagnosis.

**Results:** We identified 1,470 patients with complete data for analysis including 1,096 (74.6%) patients with AC and 374 (25.4%) patients with SCC. A total of 2,243 sites of distant metastasis were observed, the liver was the most common site of distant metastasis (727, 32.4%), followed by the distant lymph nodes (637, 28.4%), lung (459, 20.5%), bone (344, 15.3%), and brain (76, 3.4%). Multivariable logistic regression showed that compared to patients with SCC, patients with AC were more likely to have metastasis to the brain (odds ratio [OR] 3.026, 95% confidence interval [CI] 1.441-6.357, *p* = 0.003) and liver (OR 1.848, 95% CI 1.394–2.451, *p* < 0.001), and less likely to have metastasis to the lung (OR 0.404, 95% CI 0.316–0.516, *p* < 0.001). Histological type had no effect on metastasis to the distant lymph nodes or bone.

**Conclusions:** Patients with esophageal AC are more likely to present with liver and brain metastases, and less likely to present with lung metastasis than patients with esophageal SCC.

## Background

Esophageal cancer is one of the most common malignant neoplasms (1–3). Approximately half of patients have distant metastasis at initial diagnosis and more than one-third develop distant metastases after surgery or radiotherapy. Distant metastases mostly develop within 6 months of radical treatment, and median survival after diagnosis of distant metastasis is only 5 months (4–6). Therefore, distant metastasis remains the major cause of treatment failure and death in esophageal cancer.

Squamous cell carcinoma (SCC) is the predominant histological subtype of esophageal cancer in Asian countries, whereas the incidence of esophageal adenocarcinoma (AC) has been increasing in Western countries in recent decades. The etiology, clinical features, prognosis and potential treatment response of esophageal SCC and AC differ markedly (1–7). However, it is not known whether these different histological subtypes have distinct patterns of distant metastasis. In this study, we used the Surveillance, Epidemiology and End Results (SEER) database to compare the patterns of metastasis in *de novo* stage IV esophageal SCC and AC.

## Materials and methods

We included patients from the SEER database diagnosed with esophageal cancer between 2010 and 2014. The SEER program includes information on cancer incidence, treatment and mortality for approximately 30% of the US population, and is maintained by the National Cancer Institute^1^. Patients who met the following criteria were included: (1) esophageal SCC or AC with *de novo* stage IV disease at initial diagnosis; (2) information on age, gender, race/ethnicity, tumor location, histological subtype, tumor grade, tumor (T) stage, and regional lymph node status were available; (3) data on sites of synchronous metastatic lesions available, including distant lymph nodes, bone, liver, brain, and lung. Patients without positive histology were excluded. This study was approved by the institutional review boards of the First Affiliated Hospital of Xiamen University and Sun Yat-sen University Cancer Center.

The relationship between the clinicopathological features of the patients and the sites of distant metastasis were assessed via univariate analysis using the χ^2^ and Fisher's exact probability tests. Independent prognostic factors for the sites of distant metastasis were confirmed in multivariable logistic regression analysis. Risk factors that were statistically significant in univariate analysis were entered into the multivariable logistic regression model. All statistical tests were performed using SPSS (version 21.0; IBM Corporation, Armonk, NY, USA). A *p*-value less than 0.05 was considered statistically significant in all analyses.

## Results

A total of 1,470 patients with complete data available for analysis, including 1,096 (74.6%) patients with esophageal AC and 374 (25.4%) patients with esophageal SCC, were identified. Table 1 lists the clinicopathological features of the patients. Median age at diagnosis was 63 years (range, 25–96 years). Most patients were ≥50 years-old (91.0%), male (84.0%), white race (84.6%), and had regional lymph node-positive disease (77.6%).

**Table 1:** Clinicopathological features and sites of distant metastases for the 1,470 patients with esophageal cancer.

**Variable**	**Entire cohort**	**Distant lymph nodes**			**Bone**			**Brain**			**Liver**			**Lung**		
	**n**	**No**	**Yes**	***p***	**No**	**Yes**	***p***	**No**	**Yes**	***p***	**No**	**Yes**	***p***	**No**	**Yes**	***p***
**AGE (YEARS)**																
<50	133	74 (8.9)	59 (9.3)	0.802	98 (8.7)	35 (10.2)	0.405	124 (8.9)	9 (11.8)	0.383	56 (7.5)	77 (10.6)	0.041	92 (9.1)	41 (8.9)	0.917
≥50	1,337	759 (91.1)	578 (90.7)		1,028 (91.3)	309 (89.8)		1,270 (91.1)	67 (88.2)		687 (92.5)	650 (89.4)		919 (90.9)	418 (91.1)	
**GENDER**																
Male	1,235	699 (83.9)	536 (84.1)	0.905	944 (83.8)	291 (84.6)	0.738	1,170 (83.9)	65 (85.5)	0.712	607 (81.7)	628 (86.4)	0.014	864 (85.5)	371 (80.8)	0.025
Female	235	134 (16.1)	101 (15.9)		182 (16.2)	53 (15.4)		224 (16.1)	11 (14.5)		136 (18.3)	99 (13.6)		147 (14.5)	88 (19.2)	
**RACE/ETHNICITY**																
White	1,244	698 (83.8)	546 (85.7)	0.055	947 (84.1)	297 (86.3)	0.581	1,175 (84.3)	69 (90.8)	0.147	601 (80.9)	643 (88.4)	<0.001	888 (87.8)	356 (77.6)	<0.001
Black	153	99 (11.9)	54 (8.5)		122 (10.8)	31 (9.0)		150 (10.8)	3 (3.9)		95 (12.8)	58 (8.0)		84 (8.3)	69 (15.0)	
Other	73	36 (4.3)	37 (5.8)		57 (5.1)	16 (4.7)		69 (4.9)	4 (5.3)		47 (6.3)	26 (3.6)		39 (3.9)	34 (7.4)	
**LOCATION**																
Upper	59	30 (3.6)	29 (4.6)	0.033	42 (3.7)	17 (4.9)	0.414	58 (4.2)	1 (1.3)	0.707	45 (6.1)	14 (1.9)	<0.001	34 (3.4)	25 (5.4)	<0.001
Middle	176	109 (13.1)	67 (10.5)		129 (11.5)	47 (13.7)		167 (12.0)	9 (11.8)		120 (16.2)	56 (7.7)		99 (9.8)	77 (16.8)	
Lower	1,048	604 (75.5)	444 (69.7)		814 (72.3)	234 (68.0)		993 (71.2)	55 (72.4)		467 (62.9)	581 (77.9)		763 (75.5)	285 (62.1)	
Overlapping	187	90 (10.8)	97 (15.2)		141 (12.5)	46 (13.4)		176 (12.6)	11 (14.5)		111 (14.9)	76 (10.5)		115 (11.4)	72 (15.7)	
**HISTOLOGY**																
SCC	374	218 (26.2)	156 (24.5)	0.463	287 (25.5)	87 (25.3)	0.941	366 (26.3)	8 (10.5)	0.002	250 (33.6)	124 (17.1)	<0.001	200 (19.8)	174 (37.9)	<0.001
AC	1,096	615 (73.8)	481 (75.5)		839 (74.5)	257 (74.7)		1,028 (73.7)	68 (89.5)		493 (66.4)	603 (82.9)		811 (80.2)	285 (62.1)	
**GRADE**																
G1	40	26 (3.1)	14 (2.2)	0.539	28 (2.5)	12 (3.5)	0.083	35 (2.5)	5 (6.6)	0.066	21 (2.8)	19 (2.6)	0.422	26 (2.6)	14 (3.1)	0.208
G2	545	310 (37.2)	235 (36.9)		434 (38.5)	111 (32.3)		522 (37.4)	23 (30.3)		287 (38.6)	258 (35.5)		361 (35.7)	184 (40.1)	
G3-4	885	497 (59.7)	388 (60.9)		664 (59.0)	221 (64.2)		837 (60.0)	48 (63.1)		435 (58.5)	450 (61.9)		624 (61.7)	261 (56.9)	
**TUMOR STAGE**																
T1-2	635	388 (46.6)	247 (38.8)	0.003	482 (42.8)	153 (44.5)	0.584	594 (42.6)	41 (53.9)	0.052	291 (39.2)	344 (47.3)	0.002	441 (43.6)	194 (42.3)	0.627
T3-4	835	445 (53.4)	390 (61.2)		644 (57.2)	191 (55.5)		800 (57.4)	35 (46.1)		452 (60.8)	383 (52.7)		570 (56.4)	265 (57.7)	
**REGIONAL LYMPH NODE STATUS**																
Node negative	330	274 (32.9)	56 (8.8)	<0.001	247 (21.9)	83 (24.1)	0.394	315 (22.6)	15 (19.7)	0.561	124 (16.7)	206 (28.3)	<0.001	214 (21.2)	116 (25.3)	0.08
Node positive	1,140	559 (67.1)	581 (91.2)		879 (78.1)	261 (75.9)		1,079 (77.4)	61 (80.3)		619 (83.3)	521 (71.7)		797 (78.8)	343 (74.7)	

*AC, adenocarcinoma; G1, well-differentiated; G2, moderately differentiated; G3, poorly differentiated; G4, undifferentiated; SCC, squamous cell carcinoma; T, tumor*.

**Figure 1:**
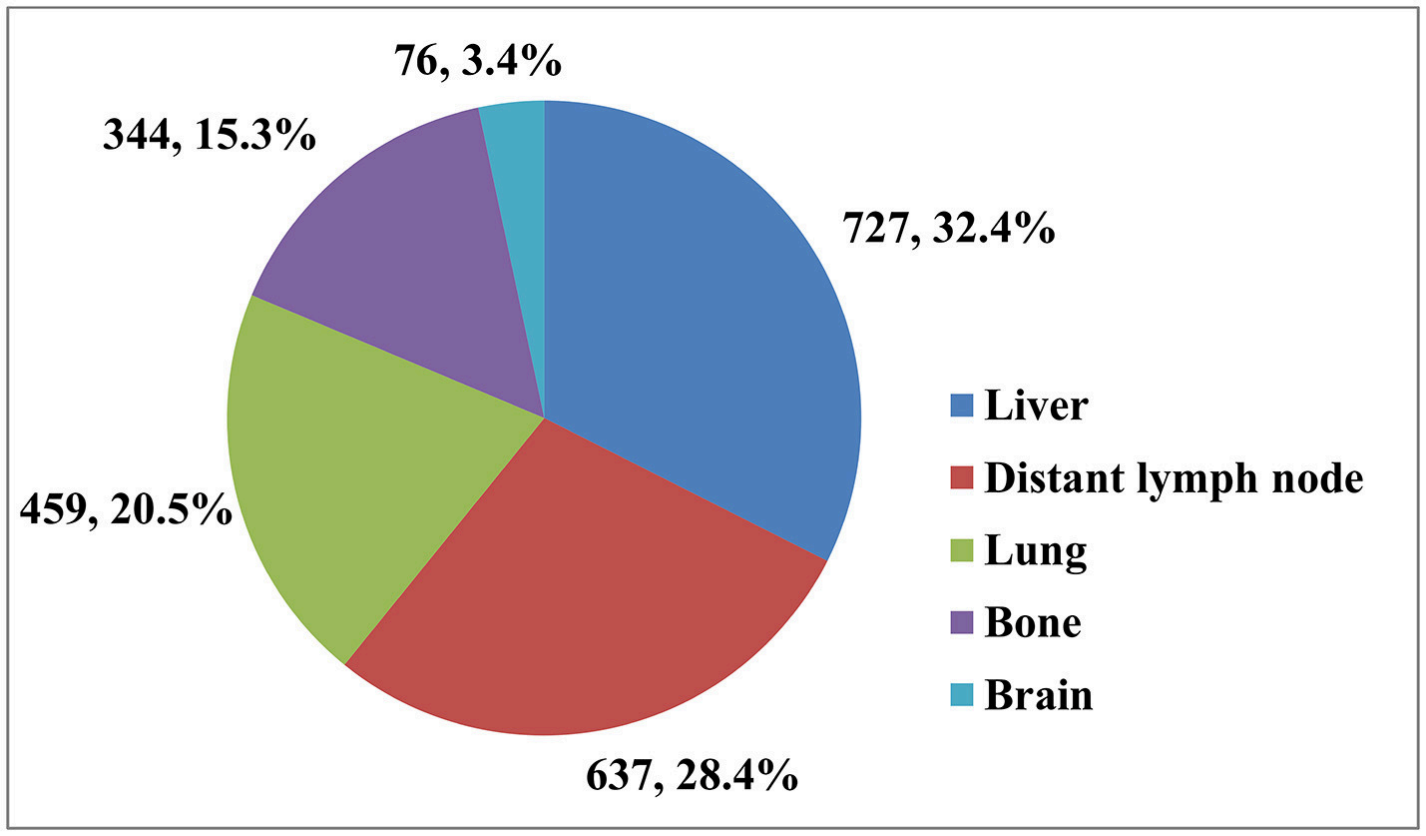
The distribution of 2,243 sites of distant metastasis in 1,470 patients.

Table 1 shows the distribution of different sites of distant metastasis for the 1,470 patients. A total of 2,243 sites of distant metastasis were observed, the liver was the most common site (727, 32.4%), followed by the distant lymph nodes (637, 28.4%), lungs (459, 20.5%), bones (344, 15.3%), and brain (76, 3.4%; Figure 1). Overall, 888 (60.4%) of patients had a single site of distant metastasis, and 424 (28.8%), 128 (8.7%), 27 (1.8%), and 3 (0.2%) patients had two, three, four, and five sites, respectively.

In univariate analysis, patients with overlapping lesions (*p* = 0.033), advanced T stage (*p* = 0.003), and regional lymph node-positive disease (*p* < 0.001) were more likely to have distant lymph node metastasis. No risk factors were associated with bone metastasis. However, patients with esophageal AC were more likely to develop brain metastasis compared to patients with SCC: of the 76 patients with brain metastases, 89.5% (*n* = 68) had AC and only eight had SCC (10.5%; *p* = 0.002). Histological subtype (*p* < 0.001), age (*p* = 0.041), gender (*p* = 0.014), race/ethnicity (*p* < 0.001), tumor location (*p* < 0.002), T stage (*p* = 0.002), and regional lymph node status (*p* < 0.002) were significantly associated with liver metastasis. In addition, histological subtype (*p* < 0.002), gender (*p* = 0.025), race/ethnicity (*p* < 0.001), and tumor location (*p* < 0.001) were associated with lung metastasis (Table 1).

**Figure 2:**
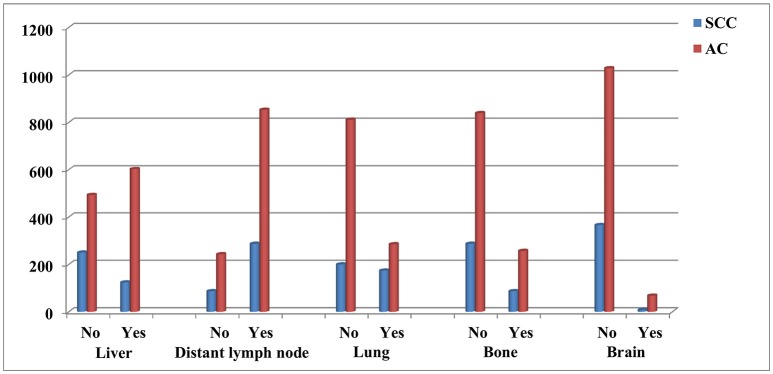
The distribution of site-specific distant metastasis by histological subtype.

The significant risk factors in univariate analysis were entered into the multivariable logistic regression model (Table 2). Regional lymph node-positive disease (odds ratio [OR] 5.071, 95% confidence interval [CI] 3.717–6.918, *p* < 0.001) was an independent risk factor for distant lymph node metastasis. Specifically, patients with AC were more likely to have brain metastasis (OR 3.026, 95% CI 1.441–6.357, *p* = 0.003), liver metastasis (OR 1.848, 95% CI 1.394–2.451, *p* < 0.001) and less likely to have lung metastasis (OR 0.404, 95% CI 0.316–0.516, *p* < 0.001) than patients with SCC. Primary tumors located in the lower esophagus were an independent risk factors for liver metastasis in esophageal cancer compared to patients with tumor located in upper esophagus (OR 0.383, 95% CI 0.199–0.739, *p* = 0.004), middle esophagus (OR 0.488, 95% CI 0.337–0.705, *p* < 0.001), and overlapping tumors (OR 0.672, 95% CI 0.482–0.935, *p* = 0.018). In addition, regional lymph node status was also the risk factor of liver metastasis in esophageal cancer. Figure 2 shows the distribution of site-specific distant metastasis by histological subtype.

**Table 2:** Multivariable logistic regression of factors associated with site-specific distant metastases in esophageal cancer.

**Variable**	**Distant lymph nodes**		**Brain**		**Liver**		**Lung**	
	**OR (95% CI)**	***p***	**OR (95% CI)**	***p***	**OR (95% CI)**	***p***	**OR (95% CI)**	***p***
**AGE (YEARS)**								
<50	—		—		1		—	
≥50	—	—	—	—	0.778 (0.536-1.129)	0.186	—	—
Gender								
Male	—		—		1		1	
Female	—	—	—	—	0.877 (0.650-1.183)	0.388	1.140 (0.840-1.545)	0.400
**RACE/ETHNICITY**								
White	—		—		1		1	
Black	—	—	—	—	0.966 (0.648-1.440)	0.866	1.247 (0.849-1.832)	0.260
Other	—	—	—	—	0.824 (0.488-1.392)	0.470	1.539 (0.933-2.539)	0.092
**LOCATION**								
Upper	1		—		1		1	
Middle	0.601 (0.322-1.120)	0.109	—	—	1.272 (0.635-2.549)	0.498	1.293 (0.703-2.378)	0.408
Lower	0.733 (0.422-1.273)	0.270	—	—	2.609 (1.354-5.026)	0.004	0.922 (0.517-1.647)	0.785
Overlapping	1.031 (0.557-1.906)	0.924	—	—	1.752 (0.878-3.495)	0.112	1.211 (0.655-2.239)	0.541
**HISTOLOGY**								
SCC	—		1		1		1	
AC	—	—	3.026 (1.441-6.357)	0.003	1.848 (1.394-2.451)	<0.001	0.404 (0.316-0.516)	<0.001
**TUMOR STAGE**								
T1-2	1		—		1		—	
T3-4	1.061 (0.848-1.327)	0.606	—	—	0.835 (0.670-1.041)	0.110	—	—
**REGIONAL LYMPH NODE STATUS**								
Node negative	1		—		1		—	
Node positive	5.071 (3.717-6.918)	<0.001	—	—	0.487 (0.376-0.6328)	<0.001	—	—

*AC, adenocarcinoma; CI, confidence interval; G1, well-differentiated; G2, moderately differentiated; G3, poorly differentiated; G4, undifferentiated; OR, odds ratio; SCC, squamous cell carcinoma; T, tumor*.

Another multivariable logistic regression model including all available variables was used to investigate the predict indicators independently associated with the histological subtype (Table 3). The results also indicated that there was an increasing incidence rate of AC subtype in patients with brain and liver metastases, younger age, male, white race, advanced T stage, and tumors located in lower esophagus compared with SCC subtype, while lung metastasis was more likely to develop in SCC subtype. The regional lymph node status, tumor grade, bone, and distant lymph node metastasis were not associated with the presence of histological subtype.

**Table 3:** Multivariable logistic regression of factors associated with the presence of histological subtype (adenocarcinoma as a reference).

**Variables**	**OR**	**95% CI**	***p***
**AGE (YEARS)**		
<50	1		
≥50	1.859	1.003-3.444	0.049
**GENDER**		
Male	1		
Female	2.591	1.763-3.806	<0.001
**RACE/ETHNICITY**		
White	1		
Black	16.326	10.161-26.233	<0.001
Other	8.106	4.466-14.712	<0.001
**GRADE**		
G1	1		
G2	1.328	0.472-3.735	0.590
G3-4	1.557	0.559-4.336	0.397
**LOCATION**		
Lower	1		
Upper	54.060	21.743-134.410	<0.001
Middle	8.445	5.557-12.833	<0.001
Overlapping	4.622	3.096-6.901	<0.001
**TUMOR STAGE**		
T1-2	1		
T3-4	0.712	0.517-0.978	0.036
**REGIONAL LYMPH NODE STATUS**		
Node negative	1		
Node positive	0.886	0.595-4.36	0.397
**DISTANT LYMPH NODE METASTASIS**		
No	1		
Yes	0.827	0.584-1.170	0.283
**LIVER METASTASIS**		
No	1		
Yes	0.559	0.406-0.769	<0.001
**LUNG METASTASIS**		
No	1		
Yes	1.832	1.331-2.521	<0.001
**BRAIN METASTASIS**		
No	1		
Ye	0.247	0.096-0.633	0.004
**BONE METASTASIS**		
No	1		
Yes	0.909	0.624-1.324	0.618

*CI, confidence interval; G1, well-differentiated; G2, moderately differentiated; G3, poorly differentiated; G4, undifferentiated; OR, odds ratio; T, tumor*.

## Discussion

Due to the high frequency of distant metastasis in esophageal cancer, it is necessary to define the patterns of spread. This study reveals site-specific patterns of distant metastasis occur in the two major histological types of esophageal cancer, indicating a need for more rigorous, tailored pretreatment imaging evaluations in each histological subtype, especially for patients with advanced stage esophageal cancer.

Similarly to previous studies that included patients with distant disease at initial diagnosis or who developed distant metastasis during follow-up (8, 9), we found the liver was the most common site of distant metastasis in esophageal cancer overall, followed by the lymph nodes, lung, bone and brain. Up to now, the patterns of distant metastasis in SCC and AC have been poorly defined. Tustumi *et al*. found patients with AC were more likely to have liver metastasis than patients with SCC (13.7% vs. 24.5%, *p* = 0.002), with no significant difference in metastasis to lung (9.8% vs. 14.8%, *p* = 0.254), bone (11.8% vs. 8.4%, *p* = 0.416) or brain (4.9% vs. 2.5%, *p* = 0.524) (9). Quint et al. also reported AC had a higher incidence of liver metastasis than SCC (37.9% vs. 9%, *p* < 0.001), with no significant differences in lung, bone, and brain metastasis (10). However, these previous studies were limited by small sample sizes and a lack of population-based data. A previous SEER study included 9,934 stage I-IV esophageal cancer patients (3,242 patients with *de novo* stage IV esophageal cancer), and the results indicated that the SCC tumors had a higher rate of lung metastasis than AC subtype, while AC subtype had a higher rate of liver, bone, and brain metastases compared with SCC tumors (11). However, the variables included in the analysis were age, gender, race/ethnicity, histological subtype, and tumor grade. The risk factors including tumor location, tumor stage, and regional lymph node status were not included in the multiple linear regression models. In addition, they also not analysis the risk factors affecting the metastasis of distant lymph nodes. In our study, we only included patients with *de novo* stage IV esophageal SCC and AC, and our results found that liver metastases were more common in patients with esophageal AC. Moreover, patients with AC presented more often with brain metastasis, whereas patients with SCC had a higher frequency of lung metastasis. Although the lymph nodes and bone are also common sites of distant metastasis, we did not observe any difference in the frequencies of distant lymph node and bone metastasis between SCC and AC. Regional lymph node status was an independent risk factor for distant lymph node metastasis, though no risk factors were associated with bone metastasis. Overall, these findings support the idea that the different histological subtypes of primary esophageal cancer exhibit distinct patterns of distant metastasis.

The underlying mechanisms driving the varied patterns of distant metastasis between the two histological subtypes are somewhat unclear. Overall, 55.0% of patients with AC and 33.2% with SCC had liver metastasis at diagnosis. Multivariate analysis demonstrated a tumor located in the lower esophagus was an independent risk factor for liver metastasis. These findings suggest the higher incidence of liver metastasis in AC may be related to tumor location as well as histological type, as most cases of AC are located in the lower esophagus, whereas SCC is more evenly distributed throughout the middle and lower esophagus.

We also observed SCC had a significantly higher risk of lung metastasis. Previous studies reported the frequency of lung metastasis was not significantly different between SCC and AC (9, 10). In a study of 35 Chinese patients with SCC and distant metastasis, 22 (62.9%) had lung metastasis and two (5.8%) had liver metastasis (12). Several studies from Japan have also reported the lung is the most common site of distant metastasis in SCC (13–15).

The brain remains a rare site of metastasis (1-5%) in esophageal cancer (16). The increased of incidence brain metastasis in recent years could be attributable to more sensitive imaging modalities or improved overall survival. Smith et al. reported that seven of 53 patients with esophageal cancer (13%) developed brain metastasis during follow-up (17). However, few studies have investigated the frequency of brain metastasis in the different histological subtypes of esophageal cancer. Studies from western countries indicate that AC accounts for 68.8–90.9% of cases of brain metastasis compared to only 9.1–31.1% for SCC (18–21). However, studies in Asian countries, including China and Japan, indicate 82.1–90.9% of cases of brain metastasis occur in SCC compared to 9.1–17.9% for AC (22–24). In this population-based study of the SEER database, brain metastases were detected in 6.2% of patients with AC and only 2.1% of patients with SCC. Of the 76 patients with brain metastases, most (89.5%) had AC.

However, no previous studies reported a significant difference in the frequency of brain metastasis between histological subtypes, which may due to their small sample sizes, especially the numbers of patients with SCC (19, 21). Previous studies reported larger primary tumors and advanced clinical stage were associated with brain metastasis (19, 21, 25). In this study, histological subtype was an independent risk factor for brain metastasis: patients with esophageal AC had a higher risk of brain metastasis than those with SCC, which may reflect the differences in tumor biology between SCC and AC. In non-small cell lung carcinoma, the incidence of brain metastasis is at least two-fold higher for AC than SCC (26–29). Due to the large differences in the distribution of the histological subtypes of esophageal cancer between Asian and Western patients, it is difficult to draw a definitive conclusion on the association between brain metastasis and histological subtype. However, biomarkers could be potentially be used to identify patients at high risk of brain metastasis in the future. For example, approximately 19–43% of cases of esophageal AC overexpress human epidermal growth factor receptor 2 (HER2), which is significantly higher than the frequency in SCC; therefore, overexpression of HER2 may potentially be associated with an increased risk of brain metastasis in esophageal AC (30–34).

It is important to describe the limitations of this study. First, retrospective studies are inherently biased. Second, the SEER database only included data on five specific sites of distant metastasis at initial diagnosis, and we could not obtain further details on the occurrence and timing of secondary metastasis. In addition, we could only extract information on synchronous metastasis to the liver, lung, bone, and brain; however, a minority of patients will develop metachronous lesions. These limitations may have led to an underestimation of other sites of metastasis, but as we have noted, the four sites of metastasis assessed in this study account for approximately 90% of metastases in stage IV esophageal cancer (8–10). Moreover, the difference in the number of patients in each subtype may affect the results, given that the SCC subtype has only one third as many patients as the AC subtype. However, the results of our study were similar to the studies from the epidemic area of SCC such as China and Japan (12, 13, 15, 22–24).

## Conclusion

In conclusion, our results suggest site-specific distant metastasis occurs in different histological subtypes of esophageal cancer. Patients with AC are more likely to develop synchronous liver and brain metastasis and less likely to develop lung metastasis than patients with SCC. Based on these differences, we suggest clinicians should take histological subtype into account when designing diagnostic and follow-up algorithms for esophageal cancer.

## Ethics statement

The study was approved by the ethics committee of the First Affiliated Hospital of Xiamen University and Sun Yat-sen University Cancer Center.

## Author contributions

S-GW, W-WZ, QL, and Z-YH are lead authors who participated in data collection, manuscript drafting, table/figure creation, and manuscript revision. W-WZ, F-YL, and J-YS aided in data collection. S-GW and W-WZ are senior authors who aided in drafting the manuscript and manuscript revision. QL and Z-YH is the corresponding author who initially developed the concept and drafted and revised the manuscript. All authors read and approved the final manuscript.

### Conflict of interest statement

The authors declare that the research was conducted in the absence of any commercial or financial relationships that could be construed as a potential conflict of interest.

## Footnotes

^1^Surveillance, Epidemiology, and End Results (SEER) Program (www.seer.cancer.gov) SEER^*^Stat Database: Incidence - SEER 18 Regs Research Data + Hurricane Katrina Impacted Louisiana Cases, Nov 2016 Sub (1973-2014 varying) - Linked To County Attributes - Total U.S., 1969-2015 Counties, National Cancer Institute, DCCPS, Surveillance Research Program, Surveillance Systems Branch, released April 2017, based on the November 2016 submission.
